# 20 Hz Steady-State Response in Somatosensory Cortex During Induction of Tactile Perceptual Learning Through LTP-Like Sensory Stimulation

**DOI:** 10.3389/fnhum.2020.00257

**Published:** 2020-06-30

**Authors:** Marion Brickwedde, Marie D. Schmidt, Marie C. Krüger, Hubert R. Dinse

**Affiliations:** ^1^Department of Neurology, BG-Universitaetsklinikum Bergmannsheil Bochum, Bochum, Germany; ^2^Neural Plasticity Lab, Institute for Neuroinformatics, Ruhr University, Bochum, Germany; ^3^Cognitive Neurophysiology Lab, Centre for Human Brain Health, School of Psychology, University of Birmingham, Birmingham, United Kingdom; ^4^Robotics Laboratory, Computer Science Institute, University of Applied Sciences Ruhr West, Mülheim an der Ruhr, Germany

**Keywords:** repetetive stimulation, tactile acuity, entrainment, alpha oscillations, phase coherence, long term potentiation, evoked potential, plasticity (A)

## Abstract

The induction of synaptic plasticity requires the presence of temporally patterned neural activity. Numerous cellular studies in animals and brain slices have demonstrated that long-term potentiation (LTP) enhances synaptic transmission, which can be evoked by high-frequency intermittent stimulation. In humans, plasticity processes underlying perceptual learning can be reliably induced by repetitive, LTP-like sensory stimulation. These protocols lead to improvement of perceptual abilities parallel to widespread remodeling of cortical processing. However, whether maintained rhythmic cortical activation induced by the LTP-like stimulation is also present during human perceptual learning experiments, remains elusive. To address this question, we here applied a 20 Hz intermittent stimulation protocol for 40 min to the index-, middle- and ring-fingers of the right hand, while continuously recording EEG over the hand representation in primary somatosensory cortex in young adult participants. We find that each train of stimulation initiates a transient series of sensory-evoked potentials which accumulate after about 500 ms into a 20 Hz steady-state response persisting over the entire period of the 2-s-train. During the inter-train interval, no consistent evoked activity can be detected. This response behavior is maintained over the whole 40 min of stimulation without any indication of habituation. However, the early stimulation evoked potentials (SEPs) and the event-related desynchronization (ERD) during the steady-state response change over the 40 min of stimulation. In a second experiment, we demonstrate in a separate cohort of participants that the here-applied pneumatic type of stimulation results in improvement of tactile acuity as typically observed for electrically applied 20 Hz intermittent stimulation. Our data demonstrate that repetitive stimulation using a 20 Hz protocol drives rhythmic activation in the hand representation of somatosensory cortex, which is sustained during the entire stimulation period. At the same time, cortical excitability increases as indicated by altered ERD and SEP amplitudes. Our results, together with previous data underlining the dependence of repetitive sensory stimulation effects on NMDA-receptor activation, support the view that repetitive sensory stimulation elicits LTP-like processes in the cortex, thereby facilitating perceptual learning processes.

## Introduction

Under everyday-live conditions, humans learn largely through practicing and repetition. In the laboratory, however, by exploiting adequate timing of pulse trains, learning can be induced merely through electrical stimulation of brain cells and synapses. The approach of repetitive sensory stimulation closes the apparent gap between these extremes by translating protocols that induce plasticity at a cellular level into sensory stimulation protocols applicable in humans.

For example, high-frequency stimulation is used to induce long-term potentiation (LTP), whereas low-frequency stimulation evokes long-term depression (LTD) (Lüscher and Malenka, 2012; Nicoll, 2017). Applying long-term potentiation-like or long-term depression-like sensory stimulation protocols to the fingertip in humans has been shown to reliably induce bidirectional changes in human perception. As a result, tactile acuity of the fingers is improved or decreased by mere exposure to stimulation, a process unaffected by confounding factors like attention or motivation (Godde et al., 2000; Ragert et al., 2008). To explain these behavioral effects, this specific form of stimulation was suggested to evoke synaptic plasticity processes in the cortical regions representing the stimulated skin sites (Pleger et al., 2001, 2003; Dinse et al., 2003). These processes can be assumed to alter synaptic transmission and remodel cortical processing, resulting in the observed behavioral changes. Evidence from studies in young adult subjects using an LTP-like protocol applied to the fingertips demonstrated major reorganization of the somatosensory cortex including changes in cortical excitability, gray matter volume, expansion of cortical representational areas, and enhanced functional connectivity between the somatosensory and motor cortex (Pleger et al., 2001, 2003; Dinse et al., 2003; Höffken et al., 2007; Heba et al., 2017; Schmidt-Wilcke et al., 2018). Most notably, it has been shown that the efficacy of repetitive sensory stimulation protocols depends on NMDA-receptor activation (Dinse et al., 2003).

While these forms of cortical reorganization imply the presence of plastic processes, up to now it remained unclear whether LTP-like protocols led to a robust and maintained repetitive activation of the somatosensory cortex, which would be the prerequisite for the induction of plastic LTP-like processes. We therefore performed continuous EEG recordings over the hand representation of the somatosensory cortex during a period of 40 min of 20 Hz intermittent stimulation of the index finger. To avoid contamination of the EEG signal with artifacts arising from electrical stimulation, we used a pneumatic device allowing reproducible air-puff stimulation of the skin (Wienbruch et al., 2006). We found that 20 Hz stimulation of the skin evoked a reliable steady-state response at 20 Hz with no indication of habituation over the range of 40 min, while sensory evoked potentials (SEPs) and event-related desynchronization (ERD) changed over time. By assessing tactile acuity measures before and after air-puff stimulation, we could show in a second experiment that the applied non-electrical stimulation also improved perception on a behavioral level.

## Materials and Methods

### Participants

The study consists of two separate experiments, performed with two independent groups of healthy, right-handed participants (experiment 1: total 15 subjects, 9 women, mean age 23.3 ± 2.9; experiment 2: total 14 subjects, 8 women, mean age 24.2 ± 3.0). Handedness of participants was confirmed using the Edinburg Handedness Inventory (Oldfield, 1971; mean laterality quotient experiment 1: 94.3 ± 9.0; experiment 2: 81.2 ± 15.7). No participant took regular medication (excluding contraceptives and in two cases thyroid hormones). In experiment 1, one participant was removed from data analysis due to poor data quality. Four participants were removed from experiment 2, as they did not reach a stable baseline in the tactile acuity task (threshold differences between baseline measures exceeded 0.15 mm). At the end of the experiment, all participants received monetary compensation. The study protocol was approved by the Ethics Committee of the Ruhr-University Bochum and in accordance with the Declaration of Helsinki. All participants provided written informed consent.

### Experimental Schedule

In the first experiment, a 4 min EEG-baseline measure was obtained which was used as a reference for later EEG-recordings. Afterward, repetitive sensory stimulation consisting of intermittent 20 Hz pulses was applied for 40 min during which participants watched an animal documentary. During the whole time, scalp EEG was continuously recorded. For the second experiment, participants first performed a baseline measure of tactile acuity on the right index finger (pre1-right). Afterward, another two baseline measures were assessed: one on the right (pre2-right) and on the left index finger (pre-left), in randomized order. Subsequently, repetitive sensory stimulation was applied for 30 min during which participants watched an animal documentary. Finally, a post measure of tactile acuity was assessed on each index finger, in randomized order (post-right and post-left).

### Repetitive Sensory Stimulation

**FIGURE 1:**
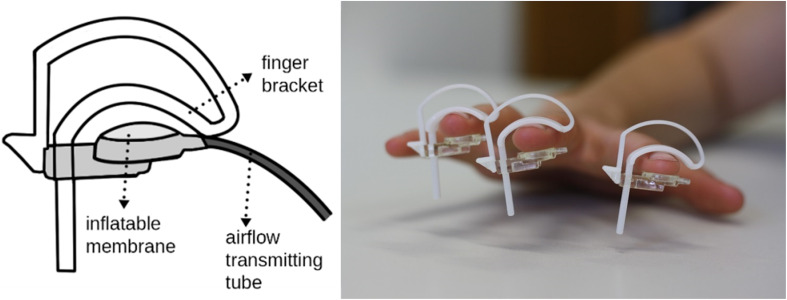
Implementation of repetitive sensory stimulation using air-puffs. Inflatable membranes, each driven by a valve controlling airflow, were applied to the index-, middle- and ring-fingers of the right hand.

In both experiments, repetitive sensory stimulation was applied to the index-, middle- and ring-fingers of the right hand using an airflow-driven membrane (Festo^®^; see Figure 1). Stimulation of three fingers was implemented to increase the efficacy of the stimulation to drive cortical activity. The stimulation protocol was the same as previously described (Ragert et al., 2008) and consisted of a 20 Hz stimulus train for 2 s, with a 5 s inter-train interval (single air-puff duration: 20 ms). The stimulation sequence was generated with a Master 8 (A.M.P.I), which forwarded TTL pulses to the EEG-trigger module (NeuroConn) as well as to the pneumatic interface (Wienbruch et al., 2006). The TTL pulses were used to control magnetic solenoid valves (operating pressure: 5 bar; Festo^®^), which were placed in an adjacent room to reduce operating noise. The airflow was transmitted to the participant using plastic standard tubes (Festo^®^), which inflated the circular membrane attached to the skin (∼8 mm^2^; 4D Neuroimaging Inc.). The latency between the Master 8 trigger output and the air-puff arriving at the membrane was 25 ms. This latency was subtracted in the presented data, so that the latency of 0 reflects the arrival of the stimulus on the skin. For a detailed description see Wienbruch et al. (2006). In experiments 1 and 2, repetitive sensory stimulation was applied for 40 and 30 min, respectively. The stimulation period of 30 min in the second experiment was chosen to clarify whether 30 min of pneumatic stimulation was sufficient to elicit tactile improvement. In the first experiment, the longer stimulation period was used to facilitate analysis of stimulus evoked oscillatory changes over time.

### EEG Recording

EEG-recordings were performed using a 13-channel DC-EEG amplifier (Thera Prax^®^ Mobile, NeuroConn) with a sampling rate of 512 Hz. Participants sat in a comfortable chair inside of a Faraday cage. Before electrode placement, the skin was cleaned with alcohol and prepared with SkinPure preparation gel. The Ag/AgCl electrodes were placed with Elefix conduction gel and arranged according to the international 10–20 system (F3, F4, CP1, CP2, PO3, PO4; ground: forehead; reference: linked mastoids). Additionally, four ocular electrodes were applied. Baseline measures alternated between two eyes-open (each lasting 1 min) and eyes-closed conditions (each lasting 30 s). Both eyes-open conditions were combined to serve as the baseline measure.

### Tactile Acuity Task

Tactile acuity was assessed on the right and left index fingertip with a modified version (Muret and Dinse, 2018) of the two-point discrimination task (2PD). It is a two-alternative forced-choice task using the method of constant stimuli (Dinse et al., 2003; Pleger et al., 2003; Ragert et al., 2008). The fingertip was placed on a custom-made device consisting of a rotatable disc with stimuli and an armrest, ensuring standardized assessment. The disc contained eight stimuli, one with a single tip and seven with two tips separated by varying distances (0.7, 1.0, 1.3, 1.6, 1.9, 2.2, and 2.5 mm). To accomplish a uniform and standardized stimulation, the disc was installed in front of an armrest that was moved up and down by the examiner. The test finger was held in a hollow containing a small hole through which the distal phalanx of the index finger was allowed to touch the probes approximately at the same indentations (about 0.5 mm) in each trial. During testing, no active movements were required from the participant (cf. Figure 1 in Schmidt-Wilcke et al., 2018). Each stimulus was presented eight times in a pseudorandomized order resulting in a total of 64 trials. Participants reported immediately after the application of the stimulus, whether they perceived one or two stimuli. Opposed to the classical task, where two tips are tested against one, participants had to differentiate between the perception of two clearly separated tips and the perception of two tips still feeling as one for smaller distances. As a marker of tactile acuity, thresholds were defined as the minimal distance with at least 50% correct identifications of two stimuli. Tactile acuity thresholds were estimated by plotting participants’ responses against needle distances and fitting them to a psychometric curve using binary logistic regression (Dinse et al., 2003; Pleger et al., 2003; Ragert et al., 2008). It should be noted that the 50% criterion is equivalent to the 75% criterion used in the GOT (grating orientation task; Johnson and Phillips, 1981), where 50% is the chance level. Both baseline measures performed on the right index finger were averaged and used as the right-hand 2PD-baseline in further analyses (test-re-test reliability was high; Cronbach’sα = 0.966).

### Data Processing and Analysis

The EEG-signal of the somatosensory electrode (CP1) was band pass filtered between 1 and 250 Hz, notch filtered around 47 and 53 Hz with a linear finite impulse response (FIR) filter, and segmented into 7 s epochs. Ocular artifacts were removed from the EEG signals using least mean squares regression (Gómez-Herrero et al., 2006). The corrected signal was manually inspected for remaining artifacts using the EEGLAB toolbox (Delorme and Makeig, 2004). In total, 12.7% of the EEG-signal was removed, indicating good data quality considering the length of trials (7 s) and the continuous recording. Sensory evoked potentials (presented in μV) were generated by computing the grand average of EEG-signal epochs over all trials and participants.

Time-frequency analyses were performed using Morlet wavelet convolution (3–60 Hz; 8–15 dynamic cycles). Resulting power values were normalized with the following formula:

(1) 10*l⁢o⁢g⁢10⁢(a⁢c⁢t⁢i⁢v⁢i⁢t⁢y/b⁢a⁢s⁢e⁢l⁢i⁢n⁢e)

where the average time interval of 700–400 ms before stimulation onset over all trials and conditions constituted the baseline.

Intertrial phase coherence values (ITPC) were derived by transforming phase angles of the complex wavelet transformation with the following formula:

(2) $$\left|n^{-1}\,\sum_{r=1}^{n}e^{i\,k\,\left(a\,n\,g\,l\,e\right)}\right|$$

Stimulus evoked power- and phase-changes from baseline were analyzed using non-parametrical permutation testing (Maris and Oostenveld, 2007). The time-frequency decomposed signal was tested against a null-hypothesis distribution, which consisted of the average of 1000 permutations, shuffled in the temporal domain. More specifically, in each iteration the time series data was shifted around a random offset. Significance was tested by applying a *T*-test to each data point and corrected for multiple comparisons using a cluster-based procedure. In this approach, the 1% largest clusters, that are adjacent data points reaching significance, were identified for each iteration. Only clusters the size or larger than the 1% largest of these clusters, were considered significant. To analyze possible changes of evoked responses over 40 min of stimulation, we compared the first and last 10 min of stimulation (the average of the first 500 ms of each stimulus train was used for this analysis). To this end, we used a similar procedure but shuffled the condition instead of the temporal domain in each iteration. Furthermore, condition differences were tested with Welch’s Test appropriate for within subject designs. The same multiple comparison correction was applied. To analyze signal processing dynamics over time more closely, we divided 40 min of stimulation into four segments of 10 min each. Frequencies and time periods of interest were analyzed using repeated measures ANOVA, correcting for multiple comparisons with Tukey-Kramer *post-hoc* tests. Finally, tactile acuity changes were analyzed using paired *T*-Tests. All statistical calculations were performed in MATLAB R2019a.

## Results

### Experiment 1

**FIGURE 2:**
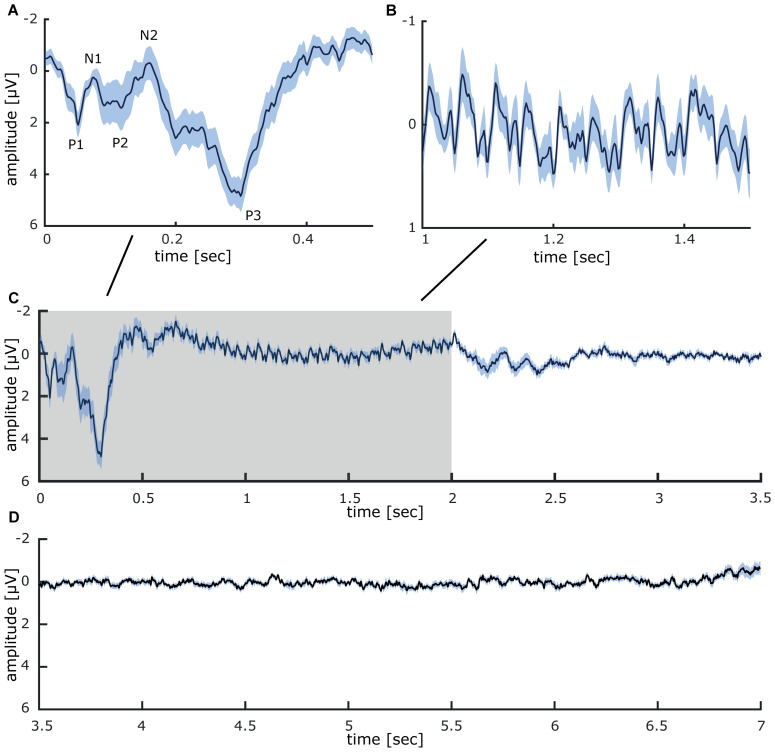
Grand average somatosensory evoked potentials measured at CP1 (international 10–20 system) during 40 min of repetitive sensory 20 Hz stimulation applied to the right index finger. **(A)** Evoked potential components recorded between onset (0 ms) and 0.5 s after stimulation. **(B)** Steady-state evoked potential between 1 and 1.5 s of intermittent 20 Hz stimulation. **(C)** Somatosensory evoked potentials in the time epoch between 0 and 3.5 s (the stimulation train interval is colored in gray). **(D)** During the inter-train interval, between 3.5 and 7 s, no stimulus-locked activity was detectible. All data is presented as mean ± SEM.

Grand average sensory evoked potentials recorded during 40 min repetitive intermittent 20 Hz stimulation of the fingertips, display clearly discernible components (presented in mean ± SEM; see Figure 2A): P1 (2.1 μV ± 0.47), N1 (0.3 μV ± 0.26), P2 (1.43 μV ± 0.87), N2 (−0.31 μV ± 0.65), and P3 (4.84 μV ± 0.62). In-between about 500–2000 ms, the 20 Hz stimulation results in a steady-state response which faithfully follows the stimulation. Two different 20 Hz components are visible, the first one with an amplitude of roughly −0.7 μV, followed by a smaller component with an amplitude of roughly −0.3 μV (see Figures 2B,C). During the inter-train interval between 2000 and 7000 ms, no clear potentials are detectible (see Figure 2D).

**FIGURE 3:**
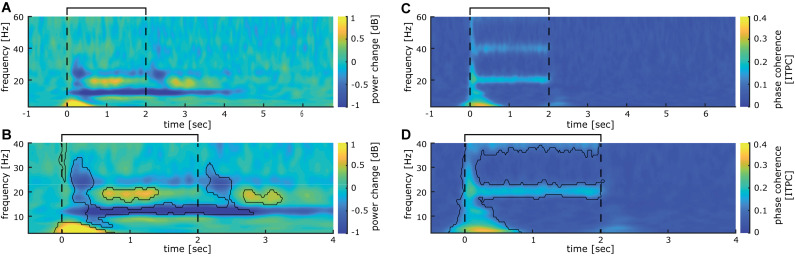
Somatosensory evoked power and phase changes during 40 min of intermittent 20 Hz sensory stimulation **(A)**, time-frequency analysis of cortical activity induced by 40 min of stimulation analyzed for the entire stimulation cycle of 7 s (2 s 20 Hz train, 5 s break) averaged over all participants. **(B)** significant clusters (*p* < 0.01; corrected for multiple comparisons with *p* < 0.01) obtained from the time-frequency analysis shown in **(A)** for the time epoch −0.5 to 3.5 s. **(C)** Inter-trial phase coherence analysis of cortical activity induced by 40 min of stimulation analyzed for the entire stimulation cycle of 7 s (2 s 20 Hz train, 5 s break) averaged over all participants. **(D)** Significant clusters (*p* < 0.01; corrected for multiple comparisons with *p* < 0.01) obtained from the inter-trial phase coherence analysis shown in **(C)** for the time epoch −0.5 to 3.5 s. The interval of the stimulation train is indicated by black lines.

Time frequency analysis of cortical responses to 20 Hz intermittent stimulation averaged across all participants revealed several significant clusters of stimulus evoked power changes (see Figures 3A,B). A strong event-related synchronization immediately following stimulation onset between 0 and 600 ms is apparent in the lower frequency range of the delta and theta band (3–8 Hz) with a maximum of 1.26 dB power change from baseline. Furthermore, between 600 and 1400 ms after stimulation onset, an event-related synchronization is visible in the 18–21 Hz range of the beta band (with a maximum of 0.54 dB power change from baseline). Interestingly, between 1600 and 2700 ms after stimulation onset, synchronization in the beta band (18–21 Hz) is visible (0.59 dB maximum power change from baseline). This activity is neither visible in the analysis of the steady-state evoked potentials (SSEPs) nor inter-trial phase coherence. Shortly after both stimulus on- (+150 ms) and offset (+500 ms), a notable event-related desynchronization (−0.73 dB maximum change from baseline) between 16 and 35 Hz occurred in the beta und low gamma band. Additionally, with a latency of 350 ms from stimulation onset, an event-related desynchronization (−1.02 dB maximum change from baseline) in the upper alpha and beta band (10–15 Hz) develops, which is maintained until the end of the stimulus train (2800 ms).

**FIGURE 4:**
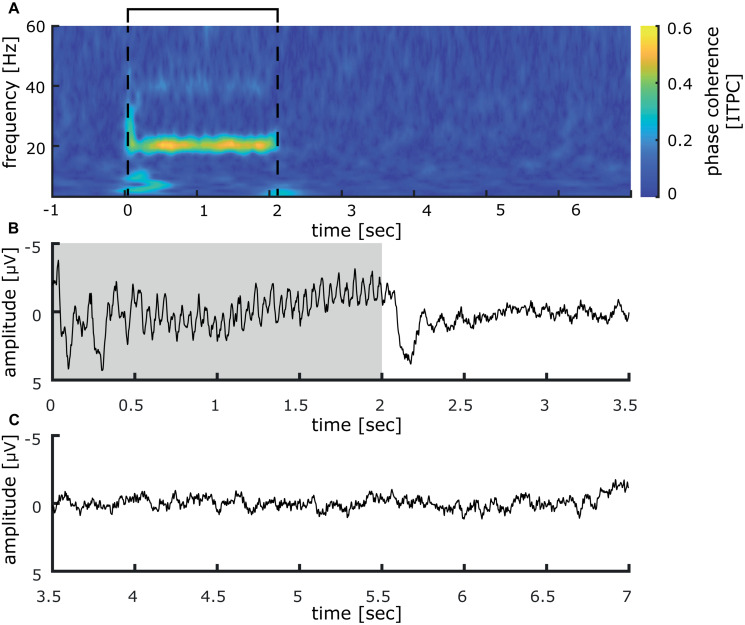
Inter-trial phase coherence analysis and somatosensory evoked potential measured at CP1 (international 10–20 system) induced by 40 min of repetitive sensory 20 Hz stimulation applied to the right index finger of one representative participant. **(A)** Inter-trial phase coherence analysis of 40 min of repetitive sensory stimulation in a representative participant illustrates varying coherence patterns between participants. The participant shows a pronounced 20 Hz phase synchronicity, while the initial responses as well as the 40 Hz synchronicity are less distinct. The interval of the stimulation train is indicated by black lines. **(B)** Somatosensory evoked potentials in the time epoch between 0 and 3.5 s. strong 20 Hz responses are visible in the EEG-signal, averaged over all trials (the stimulation train interval is colored in gray). **(C)** During the inter-train interval, between 3.5 and 7 s, no stimulus-locked activity was detectible.

Inter-trial phase coherence (ITPC) analysis over all participants revealed a large synchronous activation right after stimulation onset, which comprises all measured frequencies from 3 to 60 Hz. For the 3 Hz band, this synchronization lasts up to 850 ms, while it is reduced for higher oscillatory frequencies to around 200 ms duration (see Figures 3C,D). Most notably, a significant phase synchronicity in the 20 Hz as well as the 40 Hz band is visible for 15–2050 ms reflecting the entire duration of the 20 Hz train. In the 20 Hz range, the maximal phase synchronicity is 0.25 ITPC, while in the 40 Hz range it is 0.17 ITPC. Inter-trial phase coherence varied substantially between participants, thus weakening the effect in the grand average. This is exemplified in a representative participant shown in Figure 4, who displays a pronounced 20 Hz phase synchronicity with a maximum of 0.57 ITPC.

**FIGURE 5:**
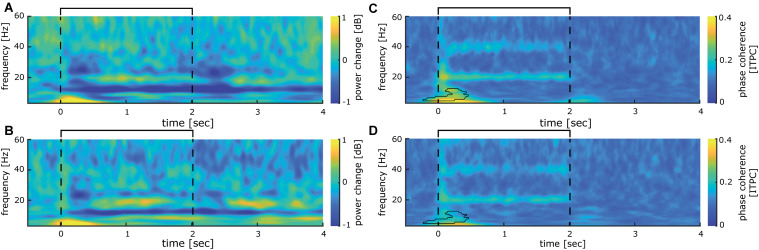
Comparison between first and last 10 min of 20 Hz intermittent stimulation. **(A)** Time-frequency analysis of cortical activity induced by the first 10 min of stimulation analyzed for the time epoch −0.5 to 3.5 s averaged over all participants. **(B)** Same as **(A)** for the last 10 min of repetitive sensory stimulation. **(C)** Inter-trial phase coherence of cortical activity induced by the first 10 min of stimulation analyzed for the time epoch −0.5 to 3.5 s averaged over all participants. **(D)** same as **(C)**, for the last 10 min of repetitive sensory stimulation. **(A–D)** The interval of the stimulation train is indicated by black lines.

To further investigate the stability of the 20 Hz cortical responses induced by repetitive sensory stimulation over time, we compared the first and the last 10 min of the 40-min stimulation period. No significant power differences could be observed between time points overall (see Figures 5A,B). The initial phase coherence at stimulation onset between -200 and 500 ms declines significantly (Figures 5C,D). Phase-locked activity during stimulation in the 20 Hz and the 40 Hz frequency bands show no sign of decline and remain stable even after 30 min of stimulation (Figures 5C,D).

**FIGURE 6:**
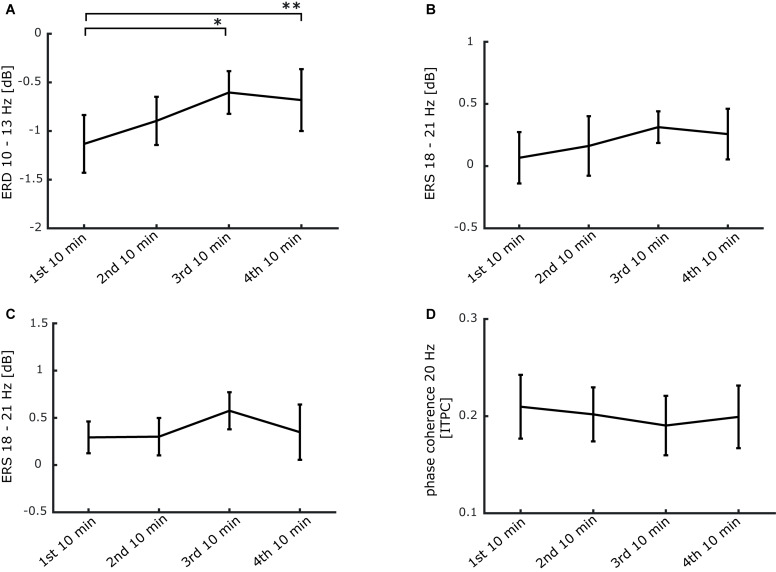
Stability of the 20 Hz steady-state response induced by 20 Hz intermittent stimulation over the stimulation period of 40 min. **(A)** ERD time course from 0.5 to 2.5 s after stimulation onset between 10 and 13 Hz (*p < 0.05; **p < 0.01). **(B)** ERS time course from 0.6 to 1.4 s after stimulation onset between 18 and 21 Hz. **(C)** ERS time course from 2.7 to 3.2 s after stimulation onset between 18 and 21 Hz. **(D)** ITPC time course from 0.5 to 2.5 s after stimulation onset at 20 Hz. **(A–D)** Data is presented as mean ± SEM.

Additionally, to monitor the time course of power and phase changes more closely, we performed repeated measures ANOVAS comparing four 10-min bins of stimulation. The ERD present from 500 to 2500 ms after stimulation onset between 10 and 13 Hz declined significantly over time (see Figure 6A). Conversely, the event-related synchronization (ERS) between 18 and 21 Hz from 700 to 1400 ms (during stimulation) as well as from 2700 to 3200 ms (in between stimulation trains) remained unchanged (see Figures 6B,C). Finally, no significant alteration was detectible in phase coherence between 500 and 2500 ms at 20 Hz (see Figure 6D). These findings indicate that the repetitive 20 Hz activity in the finger representation of SI is maintained during the entire period of stimulation with no indication for habituation.

**FIGURE 7:**
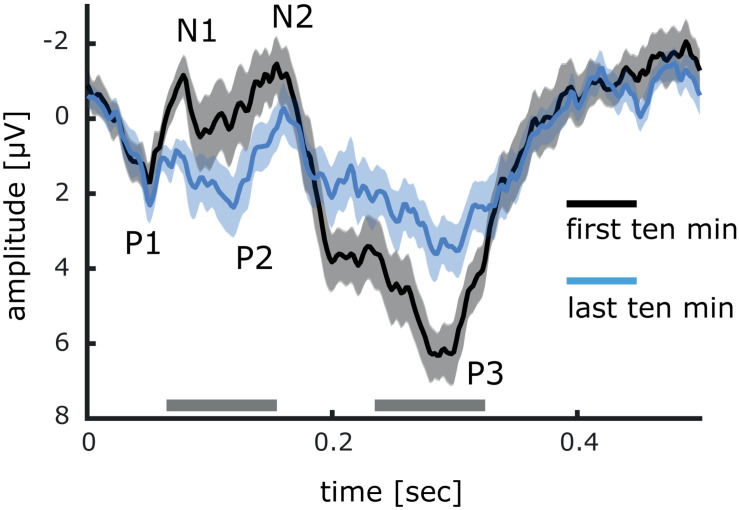
Comparison between event related potentials of the first and last minutes of 20 Hz intermittent stimulation. Differences between event related potentials have been tested with cluster permutation analysis. Significant differences *<0.05 are indicated by a gray bar. Data is presented as mean ± SEM.

To provide evidence that the early SEP components are subject to stimulation-induced changes we analyzed separately the cortical responses evoked during the first and the last 10 min of stimulation (Figure 7). Significant differences were found for the N1 (first: −1.16 μV ± 0.55; last: 0.85 μV ± 0.33) and the N2 (first: −1.46 μV ± 0.72; last: −0.28 μV ± 0.68) components, which at the end of stimulation showed less negativity. In addition, the amplitude of the P2 component was significantly enhanced (first: 0.22 μV ± 0.98; last: 2.37 μV ± 0.80). In contrast, during the last 10 min of stimulation, the amplitude of the P3 component was clearly attenuated (first: 6.32 μV ± 0.68; last: 3.60 μV ± 0.70).

### Experiment 2

**FIGURE 8:**
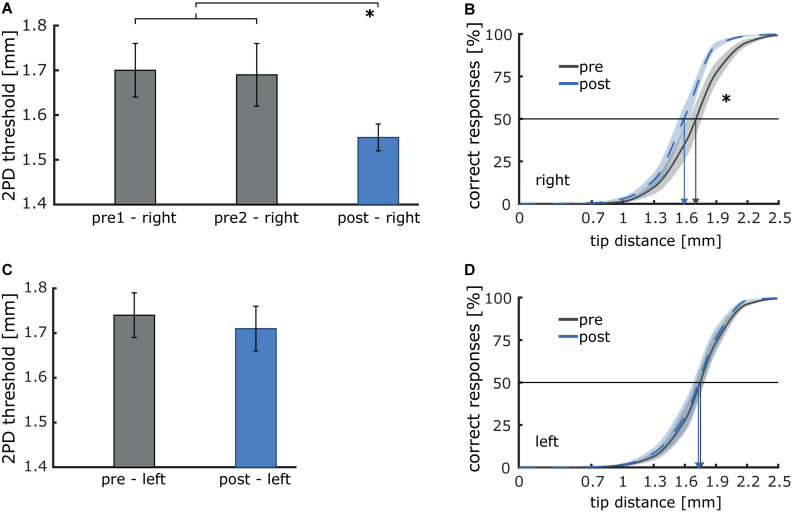
Illustration of the effect of 40 min of 20 Hz intermittent sensory stimulation on two-point discrimination (2PD) thresholds of the right and left index finger. **(A)** Discrimination thresholds before (pre1 and pre2) and after stimulation (post, *p < 0.05) for the index finger of the right stimulated hand. **(B)** Average psychometric curves pre and post stimulation. Improvement is shown by a shift of the curve to the left, arrows depict threshold values before and after stimulation. **(C)** Thresholds before (pre) and after stimulation (post) for the index finger of the left non-stimulated hand). **(D)** Average psychometric curves pre and post stimulation.

To examine, whether pneumatic air-puff stimulation induces similar effects on tactile acuity as the typically applied electrical stimulation (Ragert et al., 2008), we compared 2PD thresholds of the right index finger before and after intermittent 20 Hz stimulation. As a control condition, we tested the index finger of the left hand, which was not stimulated. On the right index finger, baseline thresholds measured during two sessions before stimulation, were stable (pre1: 1.70 mm; pre2: 1.69 mm). After stimulation, 2PD-thresholds were significantly reduced [post: 1.55 mm; *T*_(9_*_)_* = 3.18; *p* = 0.011; *d*_*z*_ = 1.01], indicating improved tactile acuity (see Figure 8). In contrast, no changes were observed on the left, non-stimulated index finger [pre: 1.74 mm; post: 1.71 mm; *T*_(9)_ = 0.80; *p* = 0.447].

## Discussion

Our results demonstrate that 20 Hz repetitive sensory stimulation delivered through air-puffs onto the fingertips induces a 20 Hz steady-state response in the hand representation of somatosensory cortex, which is maintained over a period of 40 min with no indication of habituation. In a second experiment, we could show that this type of stimulation increases tactile acuity of the fingertips to an extent comparable to electrical stimulation (Ragert et al., 2008).

Temporal processing induced by cutaneous stimulation has been studied extensively. Touch stimuli are transmitted by mechanoreceptors in the glabrous skin of the fingertip. It is likely that FA-I (fast-adapting type I) Meissner endings, sensitive to high frequency dynamic skin deformation, are involved in transmitting the 20 Hz repetitive sensory stimulation. Another type of receptors possibly contributing as well, are the FA-II (fast-adapting type II) Pacini endings, which are highly sensitive to mechanical transients and high-frequency vibrations (Johansson and Flanagan, 2009). Touch information is then transmitted via dorsal columns and the ventroposterior thalamic nuclei to the somatosensory cortex (Pons et al., 1992), where sensory evoked potentials have been recorded.

When using electrical stimulation of the median nerve, there is general agreement that the N20 component originates mainly in the granular layer (layer IV) of Brodmann’s area (BA) 3b, which occupies the posterior bank of the rolandic fissure (Allison et al., 1989, 1991; McLaughlin and Kelly, 1993; Balzamo et al., 2004). The origin of the P25 component is less clear. It has been proposed that the P25 component reflects the depolarization of the superficial portion of apical dendrites located in cortical layers 2/3 (Mitzdorf, 1985; Allison et al., 1991; McLaughlin and Kelly, 1993; Peterson et al., 1995). Other studies suggested a radially oriented source that is usually identified as BA1 at the apex of the postcentral gyrus (Arezzo et al., 1979; Allison et al., 1989; McCarthy et al., 1991). Despite these discrepancies, there is agreement that the N20 component rather reflects thalamocortical input to SI, while the N20/P25 represents intracortical processing instead (cf. Wolters et al., 2005). It should be noted that due to the cephalic channel recording used in this study, it is possible that the P25 potential is modulated by a N30 potential of frontal origin further complicating the discussion of the origin of the P25 component. Later components like P45, N60, and P/N100 are less reliable, and more susceptible to changes by cognitive factors such as attention and motivation (Michie et al., 1987; Hämäläinen et al., 1990; Ito et al., 1992; Eimer and Forster, 2003; Montoya and Sitges, 2006; Schubert et al., 2006).

Compared to electrical nerve stimulation, air-puff stimulation applied to the fingers will not alter the potential origins of the different components but will markedly modify latencies and peak properties due to less synchronized afferent activation. As illustrated in Figure 2A, a clear sequence of different components is discernible following the intermittent 20 Hz stimulation air-puff stimulation applied to the right index, middle and ring finger. However, it appears conceivable that components with a latency of 100 ms and more are confounded by the repetitive character of the stimulation, where the next incoming stimulus interacts in a complex way with the previous one (Schubert et al., 2006; Wan et al., 2008; Terrasa et al., 2018). Therefore, the following components must be interpreted with caution.

About 500 ms after stimulation onset, the evoked potentials accumulate into a steady-state response characterized by a peak at 20 Hz. Steady-state evoked potentials (SSEPs) are recorded using repetitive stimuli in a range between 5 and 50 Hz, and are analyzed using a frequency domain analysis. They elicit cortical responses, which reach maximal amplitudes in certain frequency ranges, specific for each sensory modality. In the visual system, the amplitudes of steady-state EPs are largest close to 10 and 18 Hz for unpatterned flash stimuli and at somewhat lower frequencies for patterned stimuli (Regan, 1982). In the somatosensory system, the greatest S/N ratio of the SSEP amplitudes occur at frequencies in the range of 21–26 Hz (Snyder, 1992; Tobimatsu et al., 1999; Colon et al., 2012). Accordingly, the frequency of 20 Hz used during repetitive tactile stimulation matches the preferred frequency range reported for the somatosensory cortex. Variations in preferred frequency ranges for different sensory modalities might be based on differences in the temporal characteristics of the axonal connections in a population of neurons. Moreover, the frequency corresponding to peak steady-state response was shown to inversely correlate with the size of the neural network (Hutcheon and Yarom, 2000; Colon et al., 2012; Lea-Carnall et al., 2016). On the other hand, it is controversial, whether steady-state responses are generated by superstition of independent transient responses or by neurons responding to the stimulated frequency, or both (Colon et al., 2012; Lütkenhöner, 2016). However, data from the auditory cortex favors a signal accumulating from independent transient responses (Lütkenhöner, 2016).

Using the mean phase coherence as a statistical measure for phase synchronization, we further analyzed the response properties of the evoked potentials during the intertrial phase. A first broad phase coherence covering all frequency bands most likely reflects the initial SEP components, while the following steady-state phase is reflected in the subsequent 20 Hz coherence. A less distinct, but still significant phase coherence is also apparent in the 40 Hz frequency band for the whole duration of the stimulation train, which most likely reflects the first harmonics. Repeated measures comparisons of the first 10 and the last 10 min of stimulation did not reveal significant differences in coherence, suggesting a maintained cortical steady state response without signs of habituation.

A significant event-related synchronization in the low-frequency ranges during the first 500 ms can be assumed to reflect the transient sensory evoked potentials, while the synchronization in the 20 Hz frequency range probably illustrates the steady-state response. The prominent event-related desynchronization (ERD) covering the beta and low gamma range evoked after onset and offset of the stimulation train most likely reflects the transients of the sensory evoked potentials (see Figures 2A,C). During the stimulation train, a large ERD in the alpha frequency range (10 Hz) develops about 300 ms after stimulation onset. This commonly observed response to tactile stimulation (e.g., Cheyne et al., 2003; Freyer et al., 2013) can be interpreted as a form of disinhibition to foster effective neural processing of the stimulus. Alpha oscillations have been associated with gating of information processing (Jensen and Mazaheri, 2010), thereby establishing a priority system favoring important stimuli over irrelevant information. In fact, alpha ERS before and ERD developing during repetitive sensory stimulation has recently been shown to correlate with stimulation-induced perceptual improvements, where stronger ERS before and stronger ERD during repetitive sensory stimulation were both correlated with a higher learning outcome (Freyer et al., 2013; Brickwedde et al., 2019). Additionally, non-phase-locked induced 20 Hz activity can be observed in-between stimulation trains. Such activity could reflect processing of the stimulus, which might relate to the tactile learning process.

The strength of event-related cortical responses and harmonics differed between participants. It is conceivable that differences in cortical responses during repetitive sensory stimulation are markers for effective plasticity and learning processes in the brain and only in the right combination, optimal learning conditions can be established. Therefore, future studies should analyze the effects of pneumatic repetitive sensory stimulation and perceptual learning in the same participants.

Although the ERD in the alpha band slightly declined over time, both phase coherence and stimulus evoked power in the 20 Hz range did not change over the course of stimulation. This is an important observation as it indicates that prolonged periods of identical types of stimulation will not lead to habituation of the response. The diminished ERD can be interpreted as a signature of cortical plasticity processes indicating enhanced excitability. In this case, over the period of stimulation, less and less alpha ERD is required to maintain the same level of stimulus processing. To further support this claim, we could show that the N1, N2, and P2 component of the event-related potential responses to the stimulation train was significantly altered after 20 min (Figure 7). The observed decrease of the P3 component is more difficult to interpret, because the repetitive nature of the stimulation leads to a complex mix of response behavior. In fact, repetitive sensory stimulation has previously been shown to increase cortical excitability, as paired-pulse inhibition was decreased after stimulation (Höffken et al., 2007), and the cortical BOLD response increased (Pleger et al., 2003). These changes of excitability were shown to be behaviorally relevant, as the magnitude of excitability changes correlated with stimulation-induced tactile learning.

In cellular research, long-term potentiation (LTP) and long-term depression (LTD) of synaptic transmission comprise persistent forms of activity-dependent changes in synaptic strength (Nicoll and Malenka, 1995). Typically, high-frequency stimulation is used to induce LTP in brain slices and in behaving rodents, whereas LTD can be reliably evoked by low-frequency stimulation (Bliss and Collingridge, 1993; Lynch, 2004; Malenka and Bear, 2004). Learning processes in humans are typically studied by implementing training paradigms, where inferences about underlying synaptic mechanisms are difficult. Repetitive sensory stimulation closes this gap and allows the exploration of the role and relevance of temporally structured stimulation protocols for the expression of neural plasticity processes in humans. It has been shown that 20 min of 20 Hz intermittent stimulation are sufficient to elicit cortical reorganization in form of changes to cortical representations, excitability, gray matter volume and functional connectivity (Pleger et al., 2001; Dinse et al., 2003; Höffken et al., 2007; Heba et al., 2017; Schmidt-Wilcke et al., 2018). Furthermore, on a behavioral level, tactile sensitivity on the stimulated skin site is improved (Ragert et al., 2008; Schlieper and Dinse, 2012). While it is likely, that LTP-like processes are relevant for these changes to occur, the possibility of other so far unknown processes cannot be completely ruled out. However, it has been shown that the effects of repetitive sensory stimulation, just like LTP-induction (Lüscher and Malenka, 2012; Nicoll, 2017), are NMDA-receptor dependent (Dinse et al., 2003). Furthermore, in this study we were able to show that repetitive sensory stimulation facilitates a robust and maintained activation of the somatosensory cortex, a prerequisite for LTP-like processes to occur (Nicoll, 2017). Therefore, we suggest that repetitive sensory stimulation facilitates LTP-like processes in the human somatosensory cortex, representing a fundamental tool to efficiently study conditions of plasticity processes in the human nervous system.

Some limitations of our study need to be addressed. Monitoring of potential finger flexor/extensor muscle activity could improve the signal-to-noise ratio of tactile acuity measurements, as the possibility of involuntary movement could be taken into account.

Furthermore, aiming to increase the cortical activation elicited by air-puff stimulation, we stimulated three fingers simultaneously (digit 2, 3, and 4), leading to a mix of median nerve (digit 2 and 3) as well as ulnar and radial nerve (digit 4) mediated activation. As such, comparison to more specific stimulation approaches are limited.

We have already discussed the differences between SEP characteristics obtained by median nerve and by cutaneous finger stimulation. Direct comparison is hampered by a number of factors, most critical probably the fact that median nerve stimulation is applied to the wrist, thereby activating different somatic structures as well as motor fibers, which is not the case when using finger stimulation.

Finally, it would be of great interest to combine experiment 1 and 2 in the same cohort of participants, offering the opportunity to correlate psychophysical and electrophysiological data, a direction that should be explored in the future.

## Conclusion

Our data demonstrate that repetitive stimulation using a 20 Hz protocol drives rhythmic activation in the hand representation of somatosensory cortex, which is sustained during the entire stimulation period. At the same time, cortical excitability increases as indicated by altered ERD and SEP amplitudes, while on a perceptual level, tactile acuity is improved. Our results, together with previous data underlining the dependence of repetitive sensory stimulation effects on NMDA-receptor activation, support the view that repetitive sensory stimulation elicits LTP-like processes in the cortex, thereby facilitating perceptual learning processes.

## Data Availability Statement

The datasets generated for this study are available on request to the corresponding author.

## Ethics Statement

The studies involving human participants were reviewed and approved by the Ethics Committee of the Ruhr-University Bochum. The patients/participants provided their written informed consent to participate in this study.

## Author Contributions

MB and MS carried out the experiment, performed the data acquisition, and analyzed the data. MB, MK, and HD designed the study, interpreted the data, and drafted the manuscript. All authors contributed to the article and approved the submitted version.

## Conflict of Interest

The authors declare that the research was conducted in the absence of any commercial or financial relationships that could be construed as a potential conflict of interest.
